# Investigation of the Conditions for the Synthesis of Poly(3,4-ethylenedioxythiophene) ATRP Macroinitiator

**DOI:** 10.3390/polym15020253

**Published:** 2023-01-04

**Authors:** Marin Božičević, Lucija Fiket, Magdalena Vujasinović, Roko Blažic, Marin Kovačić, Zvonimir Katančić

**Affiliations:** Faculty of Chemical Engineering and Technology, University of Zagreb, 10000 Zagreb, Croatia

**Keywords:** poly(3,4-ethylenedioxythiophene), ATRP macroinitiator, monomer reactivity, nuclear magnetic resonance, copolymer

## Abstract

One of the most widely used conductive polymers in the growing conductive polymer industry is poly(3,4-ethylenedioxythiophene) (PEDOT), whose main advantages are good thermal and chemical stability, a conjugated backbone, and ease of functionalization. The main drawback of PEDOT for use as wearable electronics is the lack of stretchable and self-healing properties. This can be overcome by grafting PEDOT with flexible side branches. As pure PEDOT is highly stable and grafting would not be possible, a new bromine-functionalized thiophene derivative, 2-(tiophen-3-yl) ethyl 2-bromo-2-methylpropanoate (ThBr), was synthesized and copolymerized with EDOT for the synthesis of a poly(EDOT-co-ThBr) ATRP macroinitiator. After the synthesis of the macroinitiator, flexible polymers could be introduced as side branches by atom-transfer radical polymerization (ATRP) to modify mechanical properties. Before this last synthesis step, the conditions for the synthesis of the ATRP macroinitiator should be investigated, as only functionalized units can function as grafting sites. In this study, nine new copolymers with different monomer ratios were synthesized to investigate the reactivity of each monomer. The ratios used in the different syntheses were ThBr:EDOT = 1:0.2, 1:0.4, 1:0.6, 1:0.8, 1:1, 0.8:1, 0.6:1, 0.4:1, and 0.2:1. In order to determine the effect of reaction time on the final properties of the polymer, macroinitiator synthesis at a 1:1 ratio was carried out at different time periods: 8 h, 16 h, 24 h, and 48 h. The obtained products were characterized by different techniques, and it was found that polymerizations longer than 24 h yielded practically insoluble macroinitiators, thus limiting its further application. Reactivity ratios of both monomers were found to be similar and close to 1, making the copolymerization reaction symmetrical and the obtained macroinitiators almost random copolymers.

## 1. Introduction

Atom-transfer radical polymerization (ATRP) belongs to the group of living or controlled polymerizations. A polymerization is considered to be living if the initiation rate is significantly greater than the propagation rate and if, in addition, termination and transfer reactions are absent [1]. Living polymerizations yield products with narrow molar mass distributions, where a dispersity (Đ, Mw/Mn ratio) of less than 1.15 can be achieved [2,3]. The active lifetime of the growing chains is “infinite” because the chains stop growing only when all the monomer is consumed. Further addition of monomer leads to additional chain growth and increase in molar masses. ATRP was first described in 1995 [4,5] and gained popularity due to its ability to control molecular weight, molecular architecture, and polymer composition at high levels and maintain low dispersity. ATRP has wide applications and is used for the synthesis of molecular brushes [6,7,8,9], stimulus-dependent polymers [10,11], self-assembly structures [12,13], and polymer copolymers with complex architecture [14,15,16]. ATRP also allows for modification of the mechanical properties of conductive polymers by grafting different amorphous side chains onto the backbone of CPs, making the generally brittle CPs stretchable and applicable in the growing field of flexible electronics [17]. Poly(3,4-ethylenedioxythiophene) (PEDOT) has emerged as one of the most promising conductive polymers for use in energy storage and wearable electronics in biomedicine due to its biocompatibility, high chemical stability, and good corrosion resistance [18,19,20,21]. To be used in flexible electronics, the stretchability of PEDOT needs to be improved. One of the possibilities is mixing PEDOT with polystyrene sulfonate (PSS), which has proven to offer increased stretchability and self-healing properties [22,23,24]. Another approach is grafting various polymer soft side chains by ATRP [25,26]. These soft side chains grafted onto the rigid PEDOT backbone make the obtained graft copolymer flexible by forming non-covalent hydrogen-bonding crosslinks. Such an approach also offers the potential advantage of obtaining a self-healing material, since hydrogen bonds can spontaneously reform as soon as they are broken by a stress [27]. Due to its high chemical stability, the PEDOT molecule does not provide reactive sites where soft side chains could be easily grafted onto the backbone. Therefore, the molecule must be modified prior to grafting. This can be achieved by copolymerizing EDOT monomer and monomer functionalized with reactive species to obtain a polymeric macroinitiator that can easily react with different side chains in the ATRP. The composition of the macroinitiator directly determines the graft density since grafting is only possible on the functionalized part of the macromolecule. Therefore, it is important to know the reactivity ratio of the monomers present in the macroinitiator so that the graft density can be tailored to the desired needs by selecting a suitable monomer mixture prior to polymerization. The reactivity ratio is a measure of the tendency of a comonomer to preferentially insert into a growing chain in which the last unit inserted was the same, rather than the other comonomer [28]. Reactivity ratios can be determined by comparing monomer feed data with copolymer composition data and then treating the data to extract the reactivity ratios that best describe the experimental data.

In this work, the EDOT monomer was copolymerized with a previously synthesized thiophene-based bromine-functionalized monomer (ThBr). Due to reactive C-Br bond presence, inclusion of ThBr provides reactive sites where different hydrogen-bonding polymer side branches could be grafted to improve stretchability and self-healability. Copolymers were obtained by chemical oxidative polymerization at various monomer feed ratios and the reactivity ratio was determined. For some commonly used copolymers, reactivity ratios can be found in the literature, so it is easy to predict the final composition of the copolymer and obtain the desired composition. As far as we know, such data cannot be found for the macroinitiator PEDOT copolymer. Therefore, these findings enable precise tailoring of grafting density.

## 2. Materials and Methods

### 2.1. Materials

All chemical reagents were used as received without any purification. 3-tiopheneethanol (97%), triethylamine (99%), 3,4-ethylenedioxythiophene (98%), silica gel, and nitromethane (99+%) were purchased from Acros Organics, Geel, Belgium. Methanol (MeOH, p.a.), dichloromethane (DCM, p.a.), ammonium hydroxide, and chloroform (CHCl_3_, p.a.) were purchased from Lach-Ner s.r.o., Neratovice, Czech Republic. Iron (III) chloride (FeCl_3_, 98%, anhydrous) was obtained from Alfa Aesar, Havernhill, MA, USA, and α-bromoisobutyril bromide (BiBB, 98%) from Tokyo Chemical Industry, Tokyo, Japan.

### 2.2. Synthetic Procedures

#### 2.2.1. Synthesis of 2-(Thiophene-3-yl)ethyl 2-Bromo2-methylpropanoate (ThBr) Monomer

**Figure 1:**
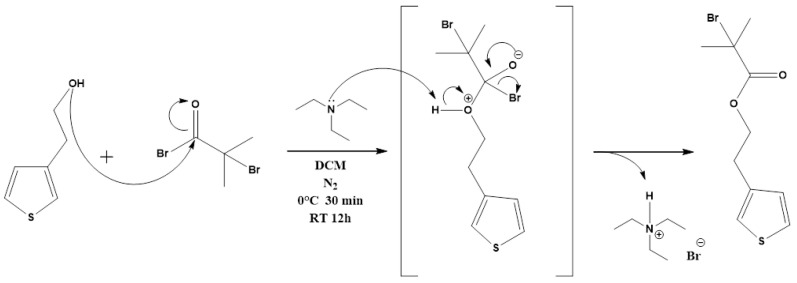
Reaction mechanism of ThBr synthesis.

ThBr monomer was synthesized according to the literature [25,29] with a few modifications in compliance with the reaction mechanism (Figure 1). A total of 100 mL of DCM was added to a 250 mL round flask at 0 °C and degassed with argon for 10 min. One equiv. of 3-thiopheneethanol (79.6 mmol, 8.74 mL) and 1.1 equiv. of triethylamine (87.56 mmol, 12.20 mL) were added and the reaction mixture was stirred for 15 min in order to activate the alcohol. A 1.1 equiv. of BiBB (87.56 mmol, 10.82 mL) was added dropwise to the reaction mixture, the reaction mixture was additionally degassed with argon, and the flask was sealed and stirred for 12 h. After 12 h of reaction, the mixture was washed three times with 100 mL of water and additionally with 100 mL of saturated aqueous solution of NaCl. Layers were separated and the organic layer was dried over MgSO_4_. The mixture was filtered and the solvent was evaporated. The residual product was then purified by column chromatography on silica gel with DCM as an eluent. Fractions containing the product were collected and concentrated in vacuo. A pale-yellow liquid was obtained as a product (21.540 g, yield = 97.6%). ^1^H NMR (600 MHz, chloroform-*d*) δ 7.28 (dd, *J* = 4.9, 2.9 Hz, 1H), 7.09–7.05 (d, 1H), 7.00 (d, *J* = 5.0, 1.3 Hz, 1H), 4.38 (t, *J* = 6.8 Hz, 2H), 3.03 (t, *J* = 6.8 Hz, 2H), 1.91 (s, 6H). ^13^C NMR (151 MHz, chloroform-*d*) δ 171.63, 137.66, 128.27, 125.67, 121.84, 65.81, 55.75, 30.77, 29.31.

#### 2.2.2. Synthesis of PEDOT-Br Macroinitiator with Different Synthesis Time

**Figure 2:**
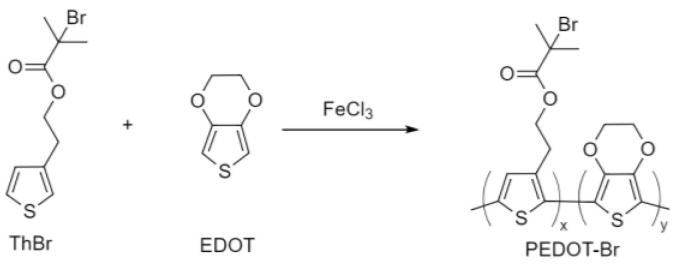
Chemical oxidative polymerization of ThBr and EDOT monomers.

The reaction scheme of the macroinitiator synthesis is shown in Figure 2. Five mL of chloroform and 1 mL of nitromethane were added to a 30 mL vial, and the mixture was purged with argon at 0 °C for 15 min. A total of 351.1 mg (2.165 mmol, 6 equiv.) FeCl_3_ was then added to the mixture with vigorous stirring. The second vial contained 4 mL of chloroform, 100 mg (0.361 mmol, 1 equiv.) of 2-(thiophen-3-yl) ethyl 2-bromo-2-methylpropanoate monomer (ThBr), and 51.3 mg (0.361 mmol, 1 equiv.) of 3,4-ethylenedioxythiophene. The mixture thus prepared was added dropwise to the FeCl_3_ solution with stirring on a magnetic stirrer and the reaction mixture was purged with argon. Simultaneously, 4 syntheses were set up with durations of 8 h, 16 h, 24 h, and 48 h. After this time, 20 mL of methanol were added to the reaction mixture to stop the reaction and precipitate the desired polymer over the next 24 h. The solution and precipitate were then transferred to centrifuge tubes and centrifuged, whereupon the solution was discarded, and the resulting precipitate was de-doped with 20 mL NH_4_OH/methanol = 1/1 for 24 h. After 24 h, the reaction mixture was centrifuged again, the solution was discarded, and the resulting precipitate was washed with methanol until the solution was decolorized. The resulting product was dried overnight at 60 °C in an oven. The resulting dry precipitate was then transferred to a vial containing 20 mL of chloroform for 48 h. The solution was then separated from the precipitate to yield soluble and insoluble products. The soluble products were then evaporated to dryness, dried in an oven at 60 °C, and characterized by various techniques to determine the effect of the duration of the synthesis on the final properties.

#### 2.2.3. Synthesis of PEDOT-Br Macroinitiator with Different Monomer Ratios

Ten mL of chloroform were placed in a 50 mL glass flask and flushed with argon for 10 min. Then, to the same vial was added an amount of each monomer from Table 1 in an inert argon atmosphere. In a separate beaker, 10 mL of chloroform and 2 mL of nitromethane were added under an inert argon atmosphere at 0 °C with stirring and a certain amount of ferric chloride was added. The solution of ferric chloride was added dropwise to the monomer solution, and everything was purged with argon and sealed tightly. The oxidant-to-monomer ratio was 3:1 for all samples. The reaction mixture was then stirred for 24 h. After 24 h, 25 mL of methanol were added to the vial to precipitate the resulting products for the next 24 h. The reaction mixture was then filtered, the resulting precipitate was taken into a vial containing 20 mL of NH_4_OH/methanol = 1/1, and the resulting mixture was stirred for 24 h. The mixture was then filtered and the precipitate was washed with a small amount of methanol, dried, and transferred to a 20 mL vial containing chloroform to separate the soluble fraction over 48 h. The resulting mixture was filtered again, and the solution was evaporated to dryness to obtain a precipitate of the soluble fraction. This precipitate was washed with diethyl ether to remove all monomer and oxidant residues. The products obtained were additionally dried at 60 °C and sent for nuclear magnetic resonance to determine the monomer ratios and the presence of impurities. The obtained samples were denoted as “TE,” which stands for “ThBr” and “EDOT,” respectively. For comparison, pure PEDOT and pure PTHBr were synthesized under the same conditions for 24 h.

### 2.3. Characterization and Instruments

#### 2.3.1. Thermogravimetric Analysis (TGA)

Thermogravimetric analysis was performed to determine the thermal stability and difference in thermal properties between samples using the TA Instruments Q500 (TA Instruments, New Castle, DE, USA). Measurements were performed under an inert nitrogen atmosphere at a flow rate of 60 mL min^−1^. The heating rate was 10 °C min^−1^ in the range of 25 °C to 600 °C.

#### 2.3.2. Gel Permeation Chromatography (GPC)

PEDOT-Br and PEDOT-*g*-PEG-MA samples were prepared by dissolving ~5 mg of the samples in 375 mg of tetrahydrofuran (THF) (1:75 ratio) and injected into the device. The injection volume of the sample was approximately 130 μL. Measurements were performed at 25 °C using a PL-GPC 20 PolymerLaboratories chromatography instrument (PolymerLaboratories, Shropshire, UK) equipped with a refractometric sensor. The separation unit consisted of two PLgelMixed-B columns connected in series and filled with poly(styrene/divinylbenzene) terpolymer gel with a particle size of 3–100 μm and THF as solvent with a flow rate of 1 cm^3^ min^−1^. The calculation of the distribution of molecular masses is based on the specific calibration curve for polystyrene.

#### 2.3.3. Fourier-Transform Infrared Spectroscopy (FTIR)

The Fourier-transform infrared spectroscopy (FTIR) measurements were performed with a PerkinElmer Spectrum One spectrophotometer (PerkinElmer, Waltham, MA, USA) using an Attenuated Total Reflectance (ATR) chamber with a ZnSe crystal in the measurement range of 4000–650 cm^−1^. The samples were dissolved in a small amount of chloroform, solution was dropped on the crystal, and as soon as the solvent evaporated, measurements were performed.

#### 2.3.4. X-ray Diffraction (XRD)

Powder X-ray diffraction (XRD) patterns were recorded using a MiniFlex 600 (Rigaku, Tokyo, Japan) diffractometer equipped with a copper target X-ray tube (Cu Kα = 1.54059 Å), a D/Tex Ultra 250 1D detector, and a sample-spinner attachment. The samples were ground to a powder using an agate pestle and mortar beforehand. Diffraction patterns of ThBr and PEDOT polymers were recorded in the 2θ range from 5° to 60° at a scan speed of 5° min^−1^, a step size of 0.01°, and sample rotation rate of 10 rpm. SmartLab Studio II (Rigaku, Tokyo, Japan) was used to analyze the collected data. The crystal structure of pristine PEDOT used for Rietveld refinement was adopted from the work of Kim and Brédas [30].

#### 2.3.5. Nuclear Magnetic Resonances (NMR)

^1^H spectra were recorded using a Bruker Avance III HD spectrometer (400 MHz) (Bruker, Billerica, MA, USA). Deuterated chloroform was used as a solvent, and chemical shifts (*δ*) were expressed in parts per million (ppm) and referenced to tetramethylsilane (TMS).

#### 2.3.6. Scanning Electron Microscopy (SEM)

Scanning electron microscopy (SEM) was utilized to examine the morphology of the samples using Tescan VEGA 3 microscope at 10 kV (Tescan, Brno, Czech Republic). Samples were coated with Pd/Au before examination.

#### 2.3.7. Four-Point Probe Method (4PP)

The electrical resistance (R) of the samples was measured using a Keysight 34.461 61/2-digit multimeter (Keysight, Santa Rosa, CA, USA) with a spacing between probes of 1.6 mm. The samples were prepared in the form of a pastille and the thickness of the samples was measured with a caliper. The electrical-resistance value was the average of 10 measurements on different parts of the samples. The electrical resistivity (*ρ*) was calculated according to the following equation [31]:

$$\rho =\frac{\pi *d*R}{\ln\left(2\right)}\;\left(\Omega \;\mathrm{cm}\right)$$

where *d*—thickness of the sample, *R*—electrical resistance, *ρ*—electrical resistivity.

The electrical conductivity (*σ*) was calculated as the reciprocal of electrical resistivity (*ρ*)

$$\sigma =\frac{1}{\rho }\;\left({S\;\mathrm{cm}}^{-1}\right)$$

## 3. Results and Discussion

### 3.1. Thermogravimetric Analysis (TGA)

**Figure 3:**
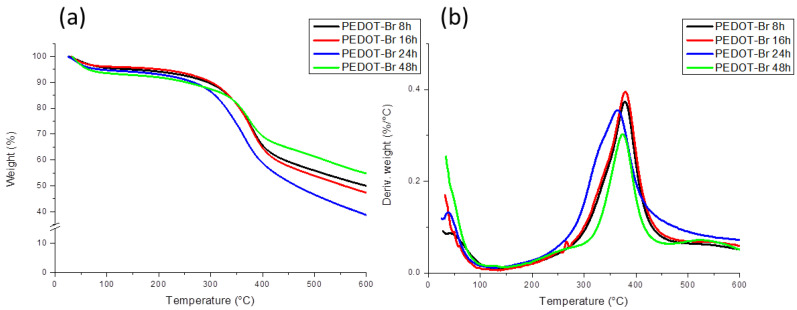
Thermogravimetric analysis of PEDOT-Br macroinitiators with different synthesis duration: (**a**) TG curves and (**b**) DTG curves of obtained products.

In order to investigate the influence of the synthesis time on the thermal stability of the macroinitiators synthesized at an EDOT:ThBr ratio of 1:1, thermogravimetric analysis was used. The results are shown in Figure 3.

**Figure 4:**
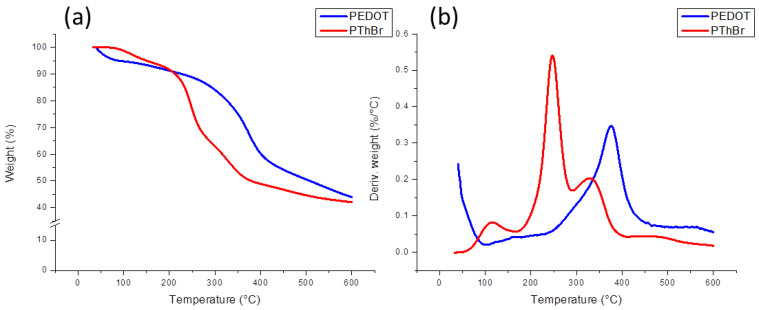
Thermogravimetric analysis of pure poly(3,4-ethylenedioxythiophene) (PEDOT) and PThBr: (**a**) TG curves and (**b**) DTG curves.

From the curves of the synthesized PEDOT ATRP macroinitiators shown in Figure 3, it is evident that the decomposition process in all four samples occurred in a single large step, starting at a temperature of about 250 °C and reaching a maximum at about 375 °C. A small weight loss of about 5% at temperatures below 100 °C was also observed, which can be attributed to the evaporation of the residual solvent. The decomposition temperatures for all samples was similar, but it can be observed that the decomposition of sample PEDOT-Br 48 h started at slightly higher temperatures; however, the maximum of the decomposition was at the same temperatures as for the other samples. This behavior could probably be due to the increase in molecular weight after 48 h of polymerization, where it took longer for the polymer molecules to degrade to small volatile fragments. No real trend was observed in the residual masses, as the lowest mass had the PEDOT-Br 24 h sample (40%), whereas the highest mass had the 48 h polymerized sample (55%) and the 8 h and 16 h samples were in between. This shows that long chains of molecules with high molecular mass were already present after 8 h of polymerization and that longer polymerization times did not have significant effects on thermal stability. For comparison, the TG results for the pure homopolymers PEDOT and PThBr polymers are shown in Figure 4. The small weight loss for PEDOT below 100 °C could again be attributed to solvent evaporation, but the main degradation steps were different for each polymer. PEDOT had one major weight loss between 250 °C and 450 °C, attributed to main chain cleaving, and the residual mass was around 45%, which was in the same range as the macroinitiator samples. Such results are in accordance with previous studies [32,33]. PThBr had lower thermal stability, with small weigh loss above 100 °C, a major degradation step at around 250 °C, and an additional step between 300 °C and 400 °C, and residual mass was again around 45%. The first small degradation step was probably the result of lower-molecular-weight oligomer degradation, followed by the removal of ethyl 2-bromo-2-methylpropanoate side branches in the main step. It can be seen from Figure 3 that macroinitiator DTG curves were closely related to pure PEDOT curves, as no weight-loss steps related to the PThBr phase were present. Such behavior could be explained by the total lack of PThBr in the copolymer, which was investigated by further methods. Other possible explanation is random placement of EDOT and ThBr monomer units in the copolymer, which would prove that the system was indeed copolymer and not just a mixture of two individual phases where the presence of ethyl 2-bromo-2-methylpropanoate side branches on ThBr instead of dioxy bridges on the PEDOT monomer had no significant effect on the thermal stability of PEDOT.

### 3.2. Gel Permeation Chromatography (GPC)

**Figure 5:**
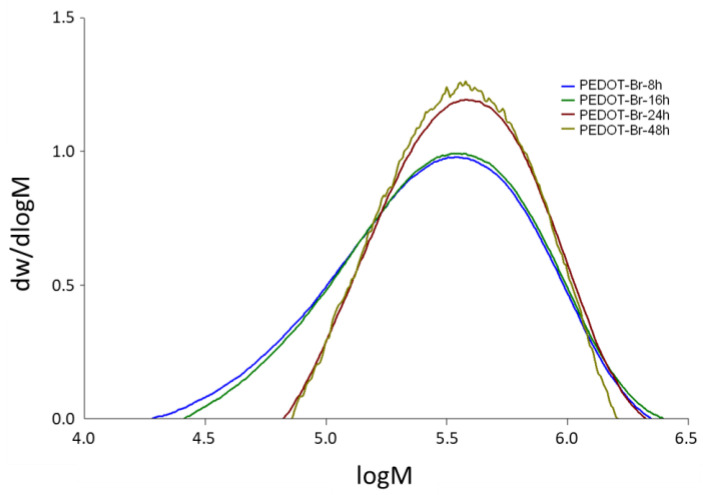
GPC analysis of PEDOT-Br macroinitiators with different synthesis durations.

From the values of molecular masses visible in Table 2, it can be concluded that an increase in synthesis time led to an increase in molecular masses. The numerical average ranged from 1.863 × 10^5^ g mol^−1^ for the 8 h synthesis to 2.941 × 10^5^ g mol^−1^ for the 24 h synthesis. After 48 h of synthesis there was no further increase in molecular mass and it remained on the same level as for 24 h. An explanation for this behavior could be that at molecular weights of about 2.90 × 10^5^ to 2.95 × 10^5^ g mol^−1^ the solubility limit of polymers in GPC analysis was reached and that all chains with higher molecular weights were insoluble. That this was indeed the solubility limit can be seen from the appearance of the curves shown in Figure 5 and was obvious when the solutions were prepared for analysis. The products from the 48 h synthesis were significantly less soluble than those from the 24 h synthesis, resulting in a much lower concentration in solution and therefore revealing noise on the curve for the 48 h synthesis. The dispersion of the samples, on the other hand, decreased with increasing synthesis time, from which it can be concluded that the polymer chains had more time to reach a similar length with longer synthesis time.

### 3.3. Fourier-Transform Infrared Spectroscopy (FTIR)

**Figure 6:**
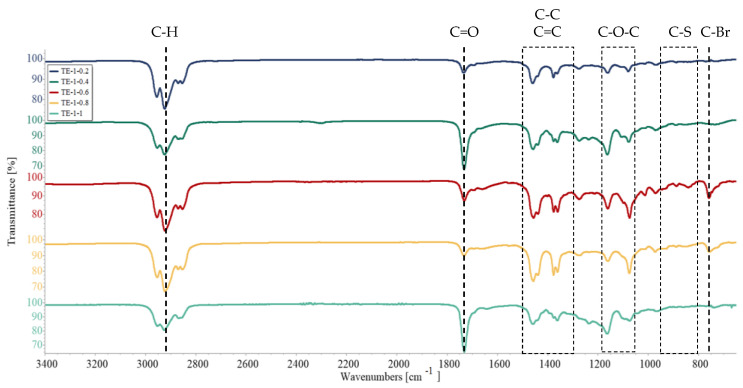
PEDOT ATRP macroinitiator FTIR spectrogram for ThBr:EDOT ratios 1:0.2; 1:0.4; 1:0.6; 1:0.8, and 1:1.

**Figure 7:**
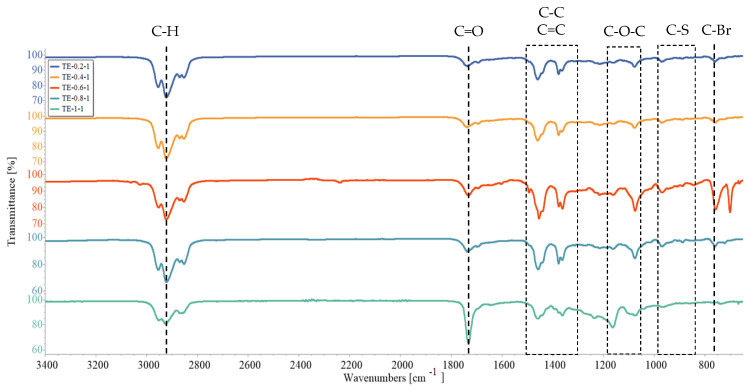
PEDOT ATRP macroinitiator FTIR spectrogram for ThBr:EDOT ratios 0.2:1; 0.4:1; 0.6:1; 0.8:1, and 1:1.

FTIR analysis was carried out for PEDOT ATRP macroinitiators obtained at different ThBr:EDOT ratios, and the results are presented in Figure 6 and Figure 7. The main difference between ThBr and EDOT monomer was the C-Br and C=O bonds present in ThBr, which were absent from EDOT. Therefore, the intensity of those bands should change correspondingly to the concentration of ThBr in the macroinitiator, being the strongest for samples TE 1:0.2 and TE 1:0.4. Nevertheless, this was not evident from the FTIR spectra. Based on the spectrograms in Figure 6 the characteristic bands were very similar for all samples, with the aforementioned bands’ intensity changing irregularly. Stretching of the C=O bond was strongest for samples TE 1:0.4 and TE 1:1, whereas the C-Br bond was strongest for samples TE 1:0.6 and TE 1:0.8. In the range of wavenumbers from 1500 cm^−1^ to 1300 cm^−1^, vibrations characteristic of the stretching of the C-C and C=C bonds in the thiophene ring were visible and the range of wavenumbers from 1200 cm^−1^ to 1000 cm^−1^ was attributed to the stretching of the C-O-C group, whereas the wavenumbers in the range from 900 cm^−1^ to 800 cm^−1^ were associated with the stretching of the C-S bond of the thiophene ring [34,35,36,37,38]. These bonds are common for both ThBr and EDOT, and therefore any change in intensity cannot be associated with the change in macroinitiator composition.

Samples shown in Figure 7 represent macroinitiator samples with increasing ThBr content. Therefore, C=O and C-Br bond intensities should be increasing. Similar to what was seen in Figure 6, there was again no observable trend. A somewhat increasing trend of C=O bond intensity at 1710 cm^−1^ was visible when comparing samples TE 1:0.2 and TE 1:1, but the same was completely missing for the C-Br bond, whose band was strongest for sample TE 1:0.6. The FTIR technique confirmed successful synthesis of the PEDOT macroinitiator but did not yield useful information about its composition, possibly due to steric hindrance of long polymer molecules. Therefore, NMR was employed for deeper investigation of the composition.

### 3.4. X-ray Diffraction (XRD)

**Figure 8:**
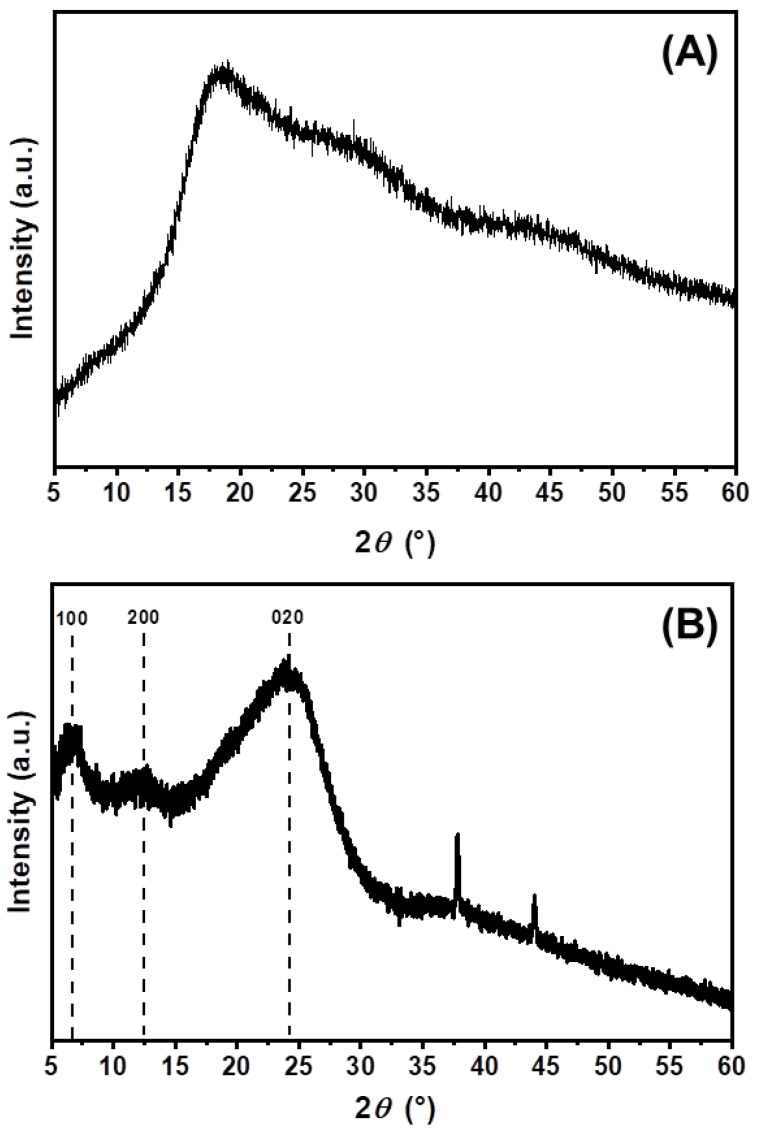
Powder X-ray diffraction patterns of (**A**) PThBr and (**B**) PEDOT.

The diffraction pattern of PThBr, shown in Figure 8A, reveals that PThBr was amorphous, as no diffraction reflections were observed. On the other hand, the diffraction pattern of PEDOT, shown in Figure 8B, indicates that the sample was semi-crystalline, as weak diffraction peaks of the crystalline regions of PEDOT were detected at 2*θ* = 6.77°, 12.45°, along with the halo evident from 2*θ*~15° to ~30° with a maximum at 24.4°. The broad halo, along with low-intensity reflection intensities, implies that the majority of the PEDOT sample was amorphous. The relatively sharp peaks observed at 2*θ* = 37.8° and 44.0° correspond to aluminum originating from the sample holder due to X-ray penetration of the sample layer [39]. The positions of the peaks corresponding to PEDOT can provide information about the dimensions of the unit cell, although Rietveld refinement was difficult due to the low signal-to-noise ratio. Therefore, the unit-cell parameters could be inferred from Bragg’s law and by analogy with the work of Aasmundtveit et al. [2]. Thus, the unit-cell parameters were assumed to be *a* = 13.0 Å, *b*/2 = 3.7 Å (*b* = 7.4 Å), and *c* = 7.1 Å. The unit-cell parameter *a* was notably smaller than reported elsewhere [40,41,42], which may be due to a lack of dopants or polystyrene sulfonate during the synthesis. The distance of the PEDOT polymer motif, i.e., the *c* unit-cell parameter in the chain, was somewhat less than reported elsewhere in the literature as well. However, the π-π stacking distance corresponding to the *b/2* parameter was somewhat larger [40,41,42], presumably due to greater charge density and hence larger contribution of repulsive electrostatic interactions.

### 3.5. Nuclear Magnetic Resonance (NMR)

**Figure 9:**
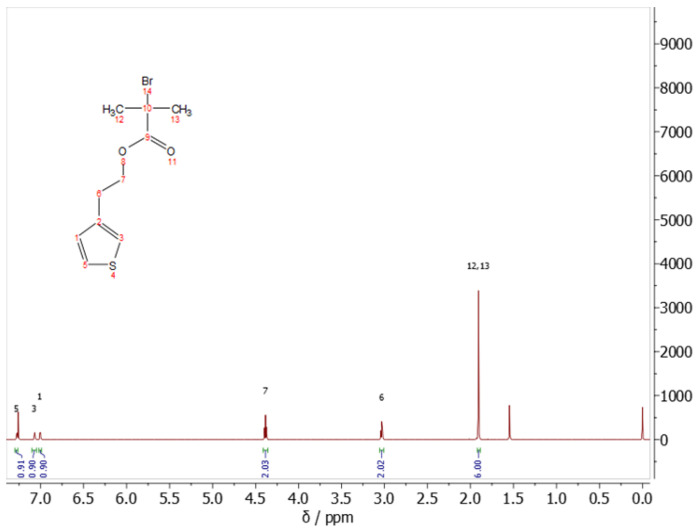
^1^H NMR spectrum of ThBr monomer.

The obtained ^1^H NMR spectrum (Figure 9) showed characteristic signals for the ThBr monomer. Three signals, 1, 3 and 5, were visible in the aromatic part and signals 6, 7, 12, and 13 in the aliphatic part of the spectrum. Two doublets at positions 1 and 3 and a doublet of doublets at position 5 of the aromatic part of the spectrum, all with an integral value equal to one, indicated the hydrogens of the thiophene ring of ThBr. The triplets at positions 6 and 7 with an integral value of two and a singlet at position 12, 13 with an integral of six could be assigned to the hydrogens of the aliphatic part of the compound. In addition to the standard ^1^H NMR spectrum, a COSY spectrum (Figure S1) was also recorded. It can be unambiguously confirmed that the arrangement of protons corresponded exactly to that suspected from the ^1^H NMR spectrum, based on the visible interaction of protons in positions 6 and 7; protons in positions 12 and 13; protons in position 1, 3 and 5; and a weak interaction between protons in positions 3 and 6.

In addition to the assignment of the hydrogen atoms, the assignment of the carbon atoms of the structure of ThBr was also carried out and shown in the ^13^C NMR spectrum (Figure S2), from which it is evident that the number of signals of the carbon atoms, 10 in number, corresponded to their number in the structure of the compound. Moreover, the values of the chemical shifts corresponded to the characteristic shifts of the carbon in the chemical environment, so the shift at position 9 corresponded to the characteristic shift of the O=C-C-X bond; the shift at position 2 corresponded to the shift associated with the Ar-C bond; the shift at positions 1, 3, and 5 corresponded to the shift associated with the C=C bond; the shift at position 7 corresponded to the shift of the C-O bond; and the shifts at positions 6, 10, 12, and 13 corresponded to the shifts of the C-C bond seen in the structure of ThBr.

**Figure 10:**
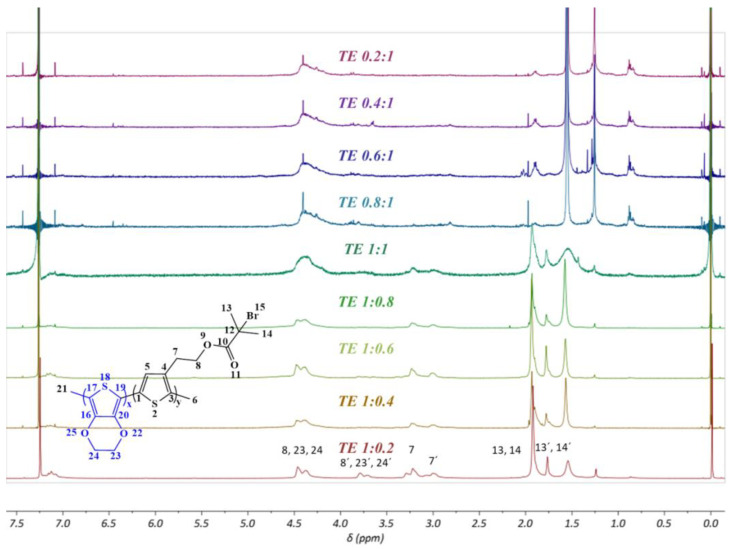
^1^H NMR spectra of products with different monomer ratios.

Figure 10 shows the ^1^H NMR spectra of all obtained PEDOT-Br macroinitiator products with different monomer ratios. To determine the real ratios and their difference from the ratio of monomers in the reaction setup, integrals of known protons belonging to specific groups of individual monomers were used (shown in the Supplementary Materials). For this purpose, a signal numbered 13 and 14 corresponding to the protons of two equal CH_3_ groups of the ThBr molecule and a signal numbered 8, 23, 24 corresponding to the protons present in the two CH_2_ groups of the EDOT molecule and one CH_2_ group of the ThBr molecule were used. If we assume that the integral of signal 13, 14 is equal to 6 because it corresponds to six protons from the two CH_3_ groups of the ThBr molecule, then we can easily determine the EDOT content in each sample from the magnitude of the integral of the signal labelled 8, 23, 24 [25]. Such a calculation yielded the ratios shown in Table 3. It can be seen that the ratios obtained agreed quite well with the original ratios, although it was obvious that a slightly higher proportion of EDOT monomers than specified was present in all samples. It can be concluded that EDOT reacts more readily and rapidly during polymerization than ThBr itself. In addition, the appearance of the double signals seen in all products with more ThBr monomer (TE 1:1–TE 1:0.2) and in the ^1^H NMR of PThBr (Figure S12) indicates that the aliphatic chains of the ThBr monomer were randomly oriented in space and that the polymers were atactic. The signal at 1.56 ppm could be assigned to water present in CDCl_3_, which is used as an NMR solvent [43].

### 3.6. Scanning Electron Microscopy (SEM)

**Figure 11:**
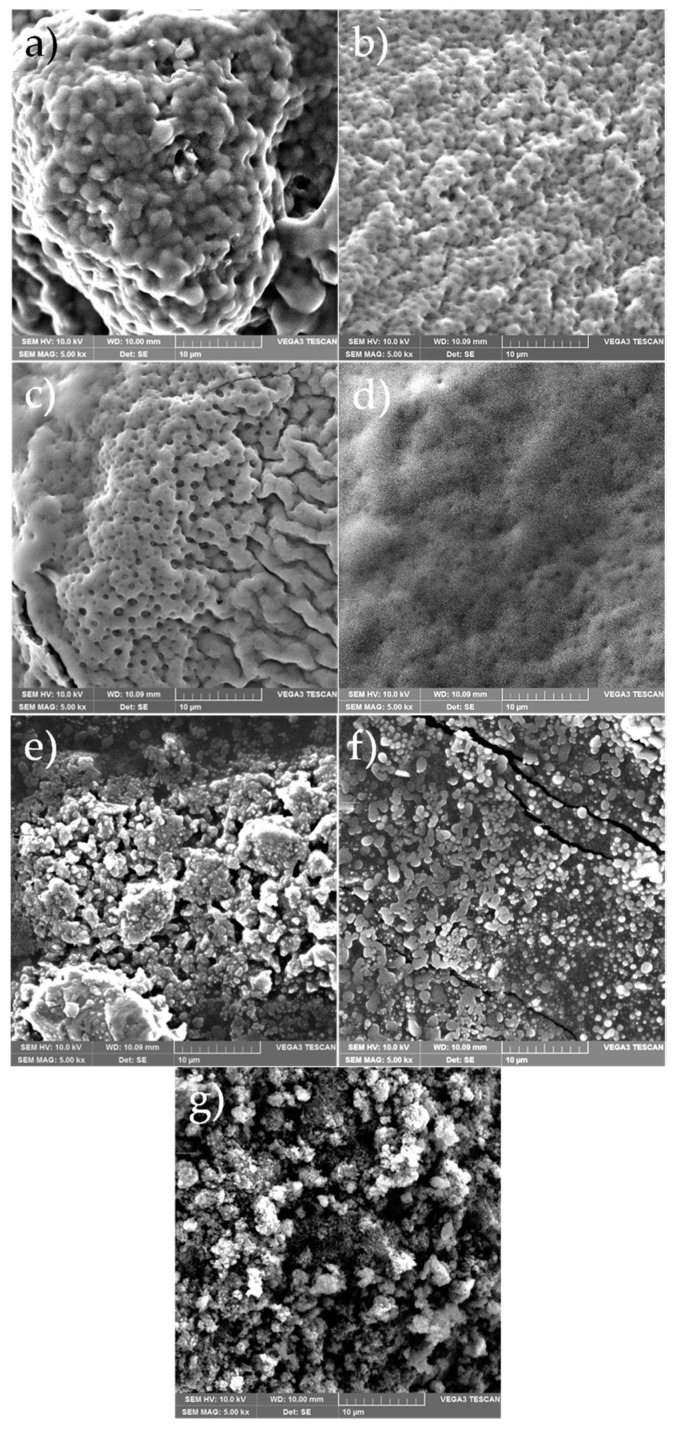
SEM pictures of sample (**a**) PThBr, (**b**) TE 1:0.2, (**c**) TE 1:0.6, (**d**) TE 1:1, (**e**) TE 0.6:1, (**f**) TE 0.2:1, and (**g**) PEDOT.

Scanning electron microscopy was used to investigate the effect of composition on the morphology of the macroinitiators studied. SEM images at 5000× magnification are shown in Figure 11. The images were taken for the samples with the highest amounts of each monomer (TE 1:0.2 and TE 0.2:1), for the samples with equal amounts (TE 1:1), for samples between the extremes and equal amounts (TE 1:0.6 and TE 0.6:1), and for homopolymers PEDOT and PThBr. The morphology changed significantly with composition. The sample with the highest ThBr content (TE 1:0.2, Figure 11b) showed a uniform amorphous morphology with small holes and voids on the surface. As the EDOT content increased, two distinct phases appeared (Figure 11c), one of which continued to be amorphous and full of voids and the other which appeared to be more regular and devoid of voids. At an equilibrium amount of ThBr and EDOT (Figure 11d), a completely uniform morphology without distinct features was obtained, although voids were observed, probably originating from the ThBr phase. When the amount of EDOT was further increased (Figure 11e,f), the morphology changed again, this time more toward particles ranging in size from a few microns to about 10 μm. It can be observed that mostly uniform but different morphologies were obtained, with the exception of the sample TE 1:0.6, where phase separation occurred.

Homopolymer PThBr (Figure 11a) had a completely amorphous structure, whereas PEDOT (Figure 11g) had visible small particles with a slightly more regular structure. This is in accordance with the XRD results wherein PThBr was found to be completely amorphous, whereas PEDOT had weak crystalline peaks suggesting a semi-crystalline structure. The morphology of the ThBr and EDOT phases was different and dominant when both were in large excess of each other. This can be attributed to the molecular structure, as EDOT has a polycyclic backbone, whereas ThBr has an aliphatic chain attached to a single thiophene ring backbone. These short side chains cause steric hindrances, which in turn affect molecular stacking in space and morphology. Nevertheless, different morphologies do not lead to significantly different thermal properties, as shown by the analysis of TG, wherein pure PEDOT was compared to the TE 1:1 sample.

### 3.7. Electrical Conductivity

As seen from the values in Table 4, the electrical conductivity decreased with increasing ThBr monomer content in the copolymers, with the lowest conductivity value for the pure PThBr sample. The electrical conductivity value of T:E 0.6:1 deviated slightly from the trend, possibly due to measurement errors, but was still very similar to the conductivity of the PEDOT homopolymer. The decreasing conductivity of the samples with higher ThBr monomer content could be a direct consequence of the aliphatic chains of the ThBr monomer, which increase the rotation of the molecule and contribute to the loss of the planar structure. According to literature [44], the conductivities of PEDOT:PSS samples range from 0.30 S cm^−1^ to 2 S cm^−1^. The values obtained in our work were lower due to the de-doping process in the synthesis of all polymers, and it can be assumed that the subsequent doping process of the prepared samples could have contributed to higher conductivities.

### 3.8. Copolymerization Reactivity

To determine the monomer reactivity ratios for the copolymerization of ThBr and EDOT, feed ratios and the compositions of the constituent monomer units in the copolymer obtained from ^1^H NMR were used. The Fineman–Ross and Kelen–Tüdos methods were performed [45] and the results are shown in Table 5. The Fineman–Ross method provides a linear correlation between *H* and *G* from which the polymer type can be determined. *H* and *G* were calculated with the following equations.

(1) $$H=\frac{f_{1}^{2}*\left(1-F_{1}\right)}{{\left(1-f_{1}\right)}^{2}*F_{1}}$$

(2) $$G=\frac{f_{1}*\left(2*F_{1}-1\right)}{\left(1-f_{1}\right)*F_{1}}$$

**Figure 12:**
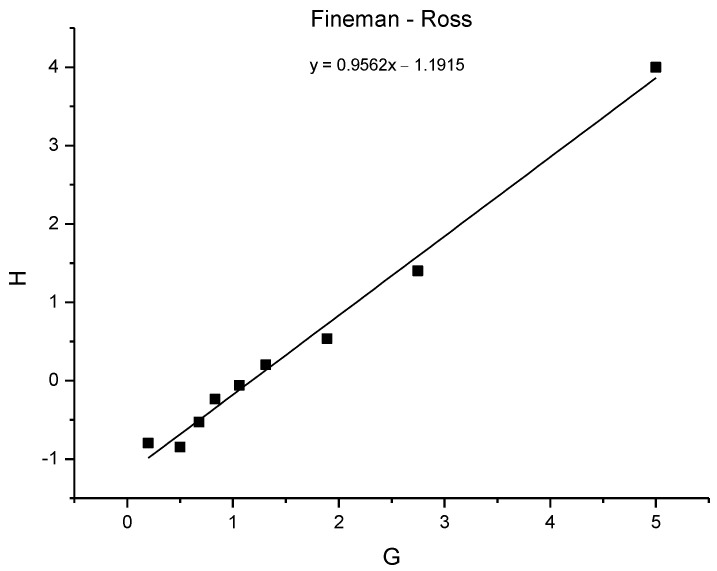
*H* versus *G* plot for monomer reactivity calculation; *r*_1_ = 0.9562, *r*_2_ = 1.1915.

A plot showing *H* versus *G* is shown in Figure 12. After linear regression, the slope of the obtained plot provides *r*_1_ and the intercept *r*_2_, where in this case *r*_1_ represents the reactivity of ThBr, whereas *r*_2_ is the reactivity of EDOT.

(3) $$G=H*r_{1}-r_{2}$$

From the Fineman–Ross plot, the monomer reactivity ratios were calculated to be *r*_1_ = 0.96, *r*_2_ = 1.19, and *r*_1_ × 2 = 1.19. Since the reactivity ratio for ThBr was slightly below 1 and that for the EDOT was slightly higher than 1, it can be concluded that the copolymers obtained were symmetric copolymers, with the EDOT monomer being slightly more reactive than the ThBr monomer, so the resulting polymer showed a higher percentage of EDOT than that found in the feed. Deviation from the ideal copolymerization was not strong, as indicated by both ratios being close to 1, and the *r*_1_ × *r*_2_ result was also close to 1. Therefore, the monomers were nearly statistically placed along the copolymer chain.

**Figure 13:**
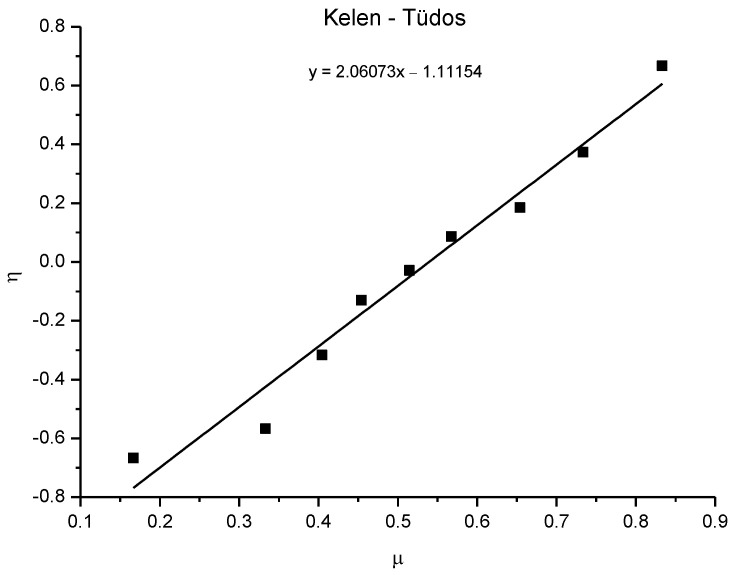
*η* versus *µ* plot for monomer reactivity calculation; *r*_1_ = 0.9492, *r*_2_ = 1.1115.

For additional confirmation of the results, the Kelen–Tüdos method [45] was performed (Figure 13). Using the *H* and *G* values obtained in the Fineman–Ross method, *α*, *η*, and *µ* were determined with the following equations to make a plot of *η* versus *µ*.

(4) $$\alpha ={\left(H_{max}*H_{min}\right)}^{0.5}$$

(5) η=G/(α+H)

(6) μ=H/(α+H)

After linear regression, the straight line provided *r*_1_ when *µ* = 1 and *r*_2_ when *µ* = 0. The values obtained by this method were similar to those obtained by the Fineman–Ross method, and it can be concluded that the polymers were obtained by symmetric copolymerization, with the EDOT monomer reacting somewhat more strongly.

This behavior allows easy adjustment of the desired composition of the macroinitiator. The density of side chains grafted by ATRP depends on the concentration of the ThBr monomer in the macroinitiator, since the only active sites where grafting is possible is the ThBr monomer. In this way, further modifications of the graft copolymer can be easily controlled by changing the length of the graft with polymerization time.

## 4. Conclusions

In this article, we report on the synthesis of conductive thiophene-based copolymer macroinitiators with different compositions. Macroinitiators are the first step in ATRP polymerizations, allowing for versatile properties and easy modifications of conductive polymers. Therefore, it is crucial to fully understand macroinitiator composition based on the starting-monomer mixture. In that way, grafting density could be easily tailored to any desired composition. Based on the FTIR and NMR data, all syntheses were successful. The Fineman–Ross and Kelen–Tüdos methods were used to determine the reactivity ratios of each monomer. Both methods yielded similar results, showing that the EDOT monomer reacted slightly faster than the Br-functionalized monomer ThBr, which yielded a copolymer macroinitiator with a slightly higher EDOT concentration than that of the starting-monomer feed. The deviation from the reactivity ratio of 1 was small, making the copolymerization reactions symmetrical and the placement of the individual monomers almost random. In addition, the effect of polymerization time on the molecular masses was investigated by GPC. The results showed that the molecular masses reached a peak value of about 300,000 g/mol after 24 h and did not increase further, indicating that the solubility limit was reached, and the products obtained by longer synthesis time had lower practical applicability due to their low solubility. Evaluation of the morphology by SEM showed a mixed morphology depending on which monomer was predominant, with the PEDOT phase slightly contributing to a more crystalline structure, also confirmed by XRD. Analysis by TG showed that thermal stability was not significantly affected by the addition of the ThBr monomer, so all macroinitiators were stable at high temperatures. Electrical conductivity decreased with ThBr content, implying that lower grafting density should be preferred to maintain conductivity.

## Figures and Tables

**Table 1:** Reactant and oxidant quantities used in different syntheses.

Sample	*m* (ThBr)/mg	*m* (EDOT)/mg	*m* (FeCl_3_)/mg
TE-1:0.2	208	21	583.9
TE-1:0.4	208	43	681.3
TE-1:0.6	208	64	778.7
TE-1:0.8	208	86	876.1
TE-1:1	200	102	930.7
TE-0.8:1	160	102	837.6
TE-0.6:1	120	102	744.5
TE-0.4:1	80	102	651.4
TE-0.2:1	40	102	558.3

**Table 2:** Gel permeation chromatography (GPC) results.

Sample Name	*Mn*	*Mw*	*Ð*
	g mol^−1^		
PEDOT-Br 8 h	1.863 × 10^5^	4.002 × 10^5^	2.15
PEDOT-Br 16 h	2.030 × 10^5^	4.169 × 10^5^	2.05
PEDOT-Br 24 h	2.941 × 10^5^	4.636 × 10^5^	1.58
PEDOT-Br 48 h	2.900 × 10^5^	4.376 × 10^5^	1.51

**Table 3:** Starting monomer ratios and final monomer ratios determined by NMR.

	Starting Ratio		NMR Ratio	
Sample	ThBr	EDOT	ThBr	EDOT
TE 1:0.2	1	0.2	1	0.20
TE 1:0.4	1	0.4	1	0.44
TE 1:0.6	1	0.6	1	0.68
TE 1:0.8	1	0.8	1	0.84
TE 1:1	1	1	1	1.06
TE 0.2:1	0.2	1	0.20	1
TE 0.4:1	0.4	1	0.32	1
TE 0.6:1	0.6	1	0.53	1
TE 0.8:1	0.8	1	0.77	1

**Table 4:** Electrochemical properties of prepared samples.

Sample Name	*R* (Ω)	*ρ* (Ω cm)	*σ* (S cm^−1^)
PEDOT	1916	722	1.39 × 10^−3^
T:E 0.6:1	2752	694	1.44 × 10^−3^
T:E 1:1	5630	1372	0.73 × 10^−3^
T:E 1:0.6	4875	3555	0.28 × 10^−3^
PThBr	20,167	6977	0.14 × 10^−3^

**Table 5:** Parameters for the statistical copolymers.

	Starting Monomer Ratio			Monomer Ratio in Copolymer			Fineman–Ross		Kelen–Tüdos	
Sample	*M*_1_	*M*_(1+2)_	*f*_1_	*M*_1*_	*M*_(1+2*)_	*F*_1_	*H*	*G*	*η*	*µ*
TE 1:0.2	1.00	1.20	0.83	1.00	1.20	0.83	5.00	4.00	0.67	0.83
TE 1:0.4	1.00	1.40	0.71	1.00	1.44	0.69	2.75	1.40	0.37	0.73
TE 1:0.6	1.00	1.60	0.63	1.00	1.68	0.60	1.89	0.53	0.18	0.65
TE 1:0.8	1.00	1.80	0.56	1.00	1.84	0.54	1.31	0.20	0.09	0.57
TE 1:1	1.00	2.00	0.50	1.00	2.06	0.49	1.06	−0.06	−0.03	0.51
TE 0.8:1	0.80	1.80	0.44	0.77	1.77	0.44	0.83	−0.24	−0.13	0.45
TE 0.6:1	0.60	1.60	0.38	0.53	1.53	0.35	0.68	−0.53	−0.32	0.40
TE 0.4:1	0.40	1.40	0.29	0.32	1.32	0.24	0.50	−0.85	−0.57	0.33
TE 0.2:1	0.20	1.20	0.17	0.20	1.20	0.17	0.20	−0.80	−0.67	0.17

* denotes the monomer ratio in copolymers.

## Data Availability

The data presented in this study are available on request from the corresponding author.

## Supplementary Materials

The following are available online at https://www.mdpi.com/article/10.3390/polym15020253/s1, Figure S1: COSY spectrum of the ThBr monomer, Figure S2: ^13^C spectrum of the ThBr monomer, Figure S3: ^1^H NMR spectrum of product TE-1:0.2, Figure S4: ^1^H NMR spectrum of product TE 1:0.4, Figure S5: ^1^H NMR spectrum of product TE 1:0.6, Figure S6: ^1^H NMR spectrum of product TE 1:0.8, Figure S7: ^1^H NMR spectrum of product TE 1:1, Figure S8: ^1^H NMR spectrum of product TE 0.8:1, Figure S9: ^1^H NMR spectrum of product TE 0.6:1, Figure S10: ^1^H NMR spectrum of product TE 0.4:1, Figure S11: ^1^H NMR spectrum of product TE 0.2:1, Figure S12: ^1^H NMR spectrum of PThBr.
