# Supplementary material for: Investigation of the Conditions for the Synthesis of Poly(3,4-ethylenedioxythiophene) ATRP Macroinitiator
Source: Polymers (Basel). 2023 Jan 4;15(2):253. doi: 10.3390/polym15020253 (PMC9867338; doi:10.3390/polym15020253)
Supplement: Supplementary file 1 [file polymers-15-00253-s001.zip › polymers-1991156-supplementary.pdf]

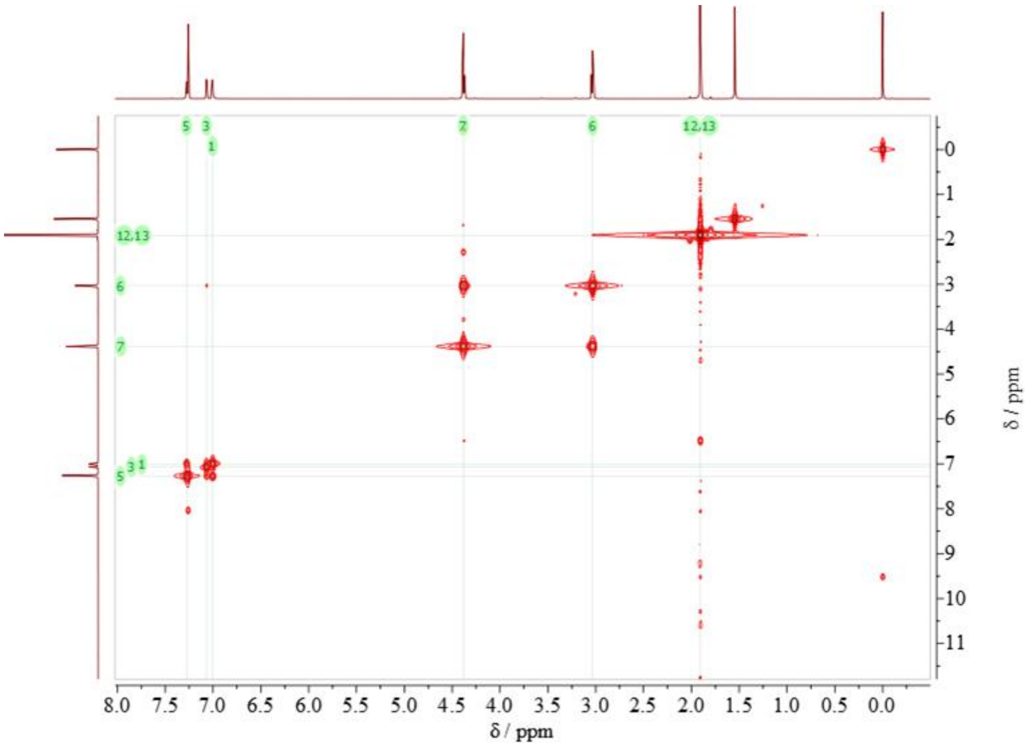

Figure S1. COSY spectrum of ThBr monomer

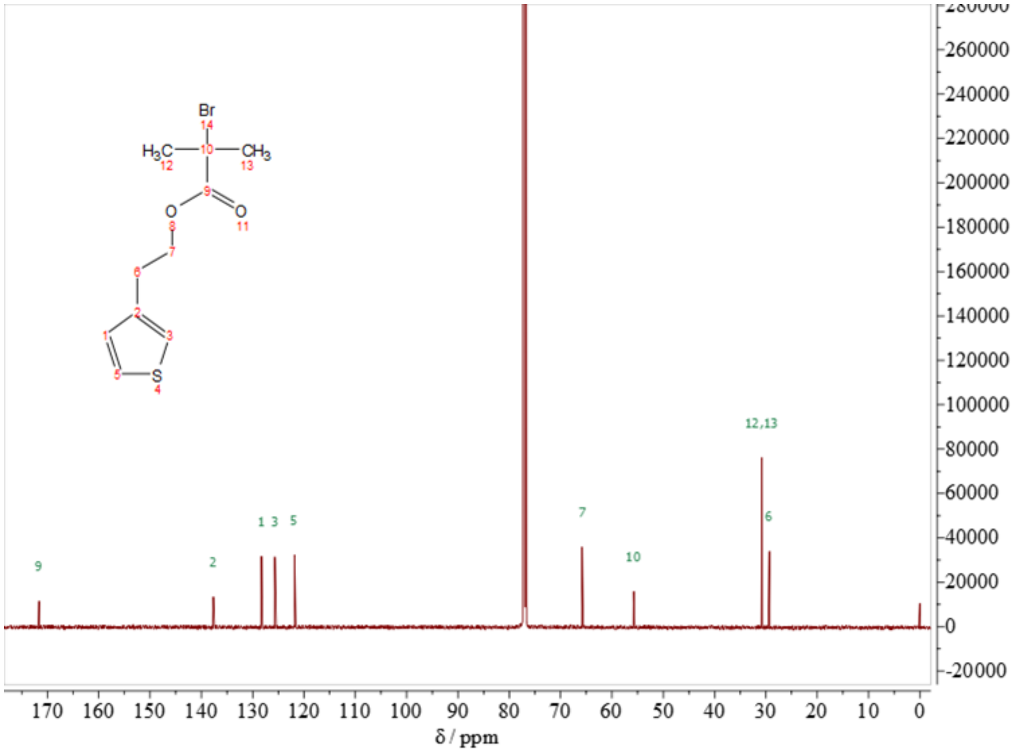

Figure S2. ^{13}C spectrum of ThBr monomer

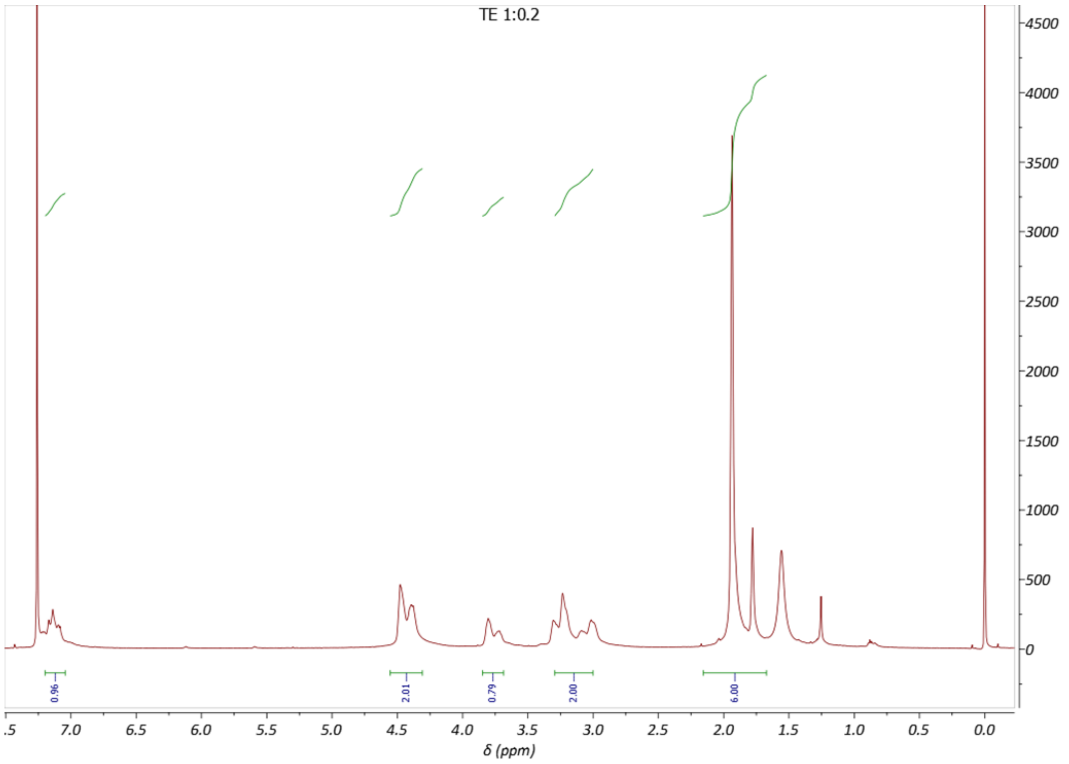

Figure S3. ^{1}H NMR spectrum of product TE-1:0.2

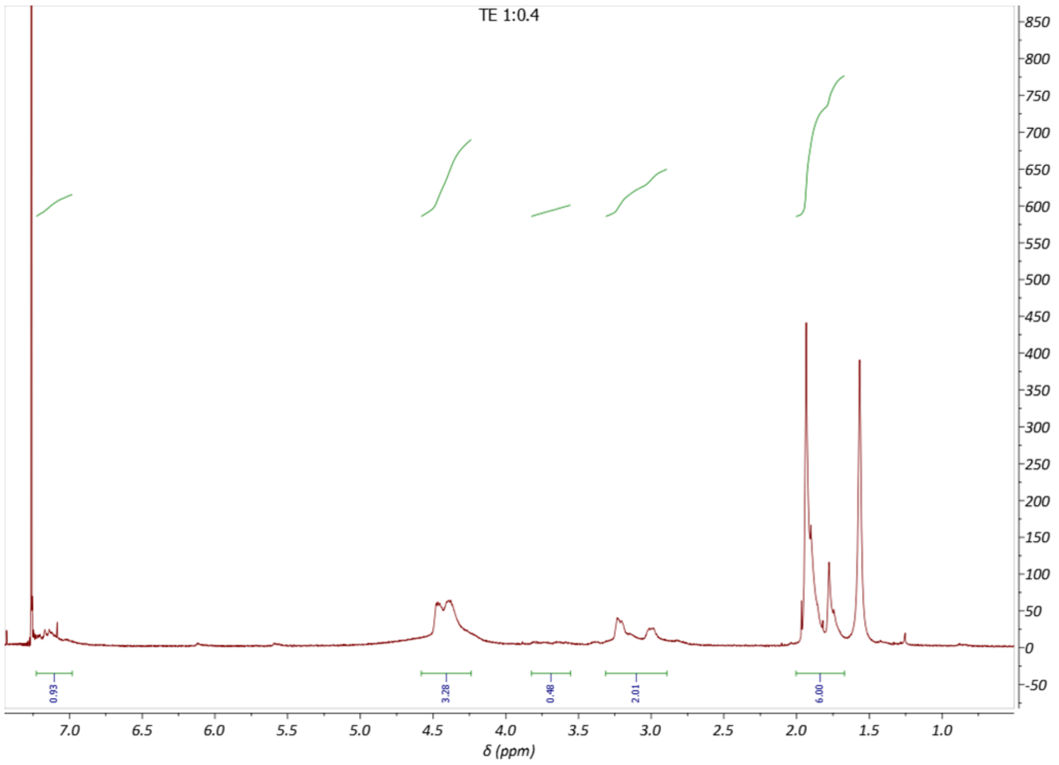

Figure S4. ^{1}H NMR spectrum of product TE 1:0.4

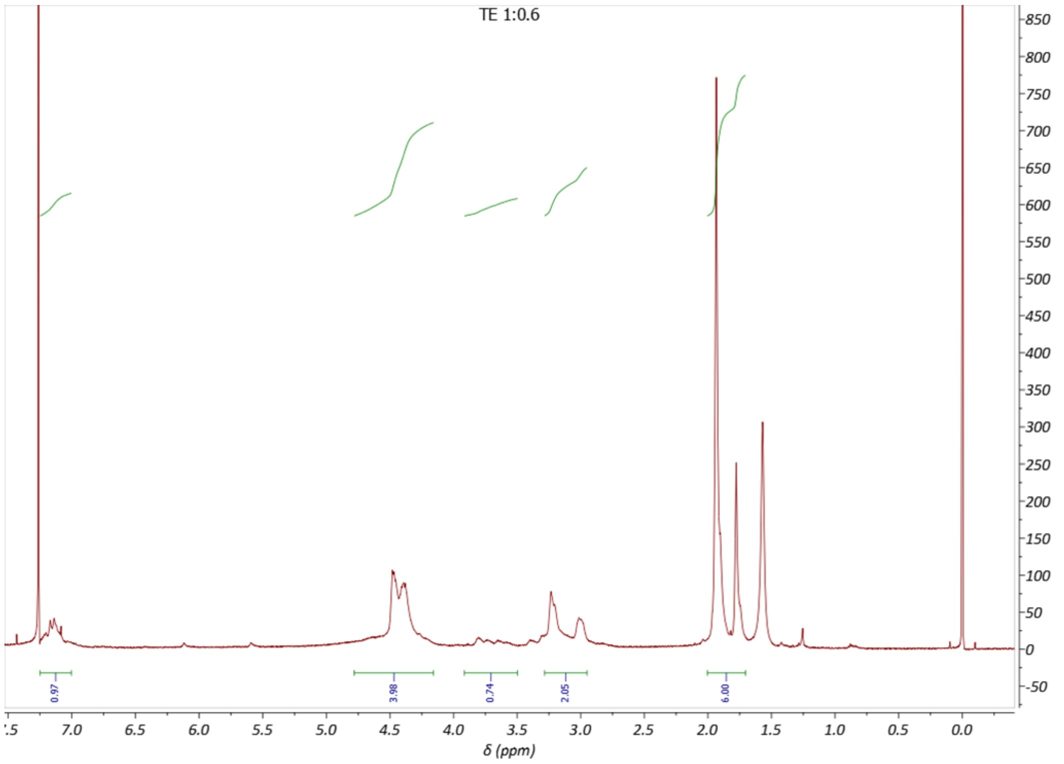

Figure S5. ¹H NMR spectrum of product TE 1:0.6

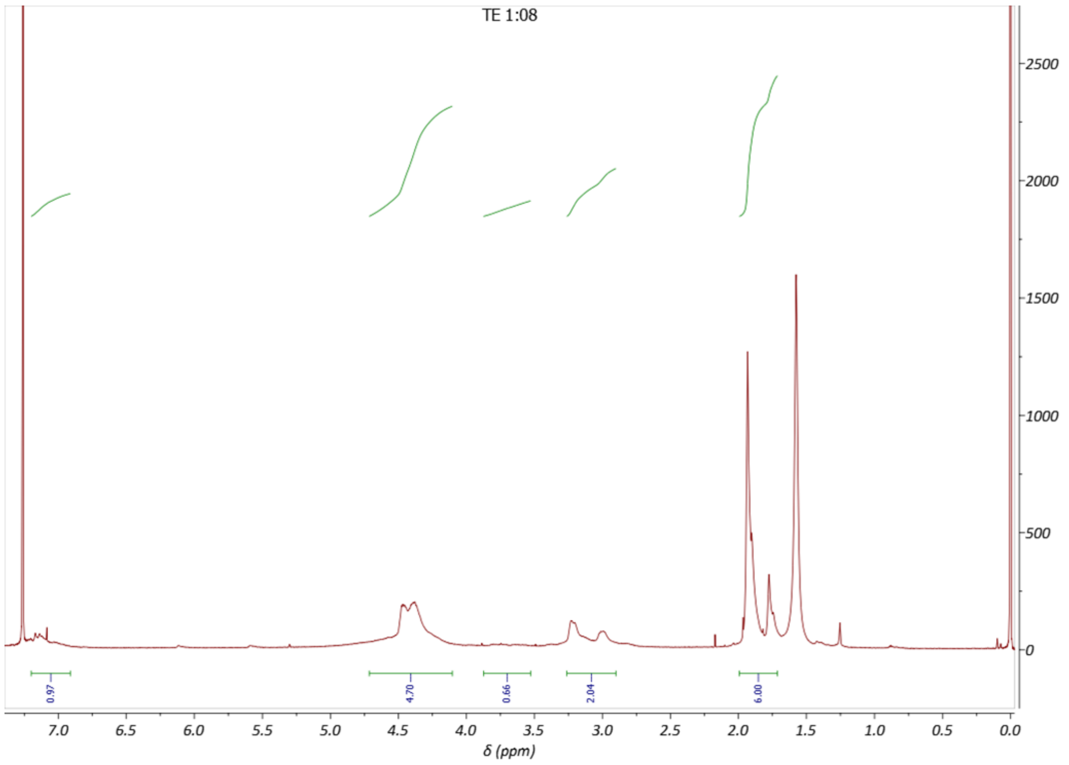

Figure S6. ¹H NMR spectrum of product TE 1:0.8

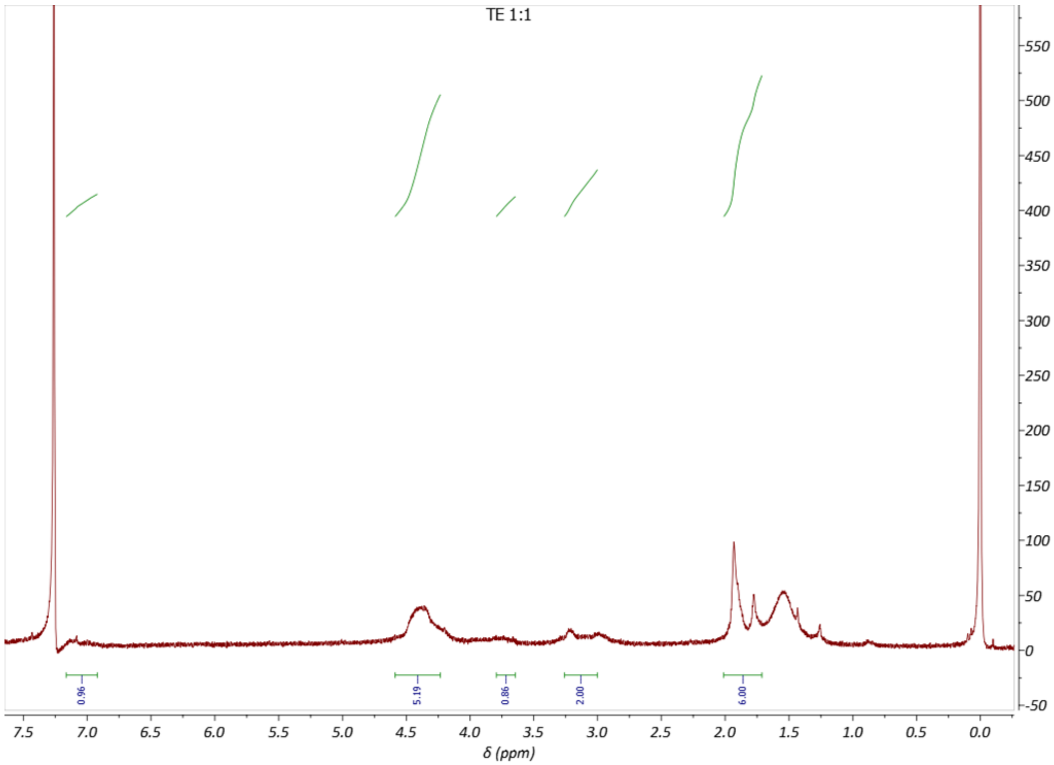

Figure S7. ^{1}H NMR spectrum of product TE 1:1

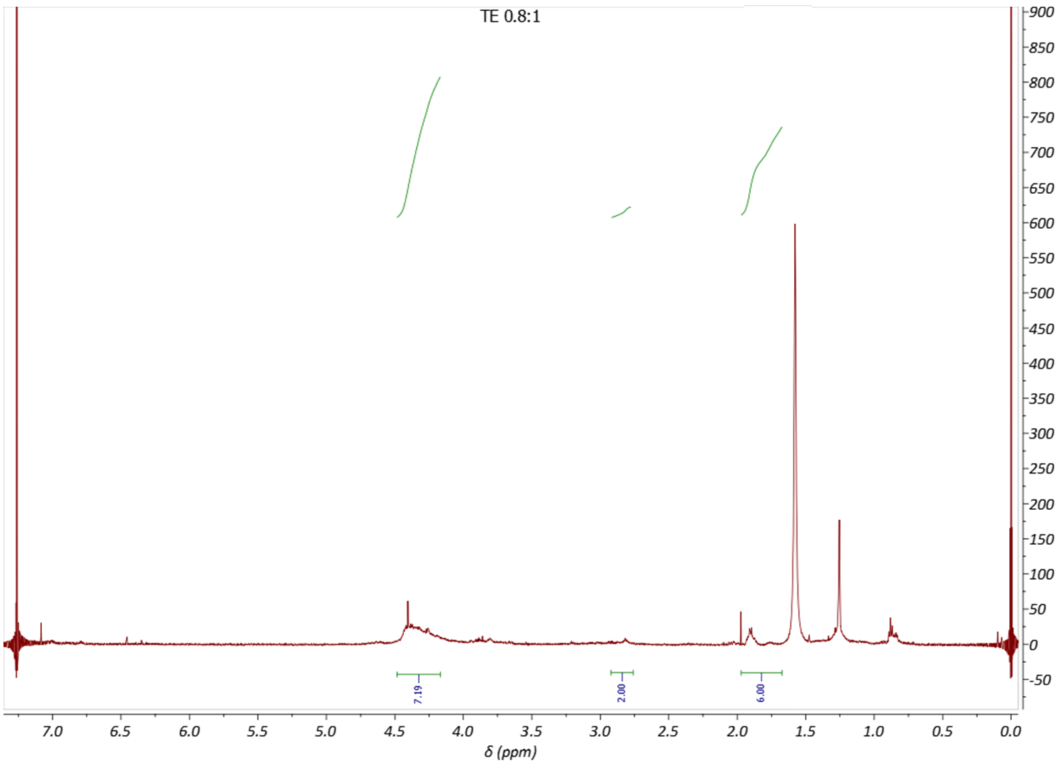

Figure S8. ^{1}H NMR spectrum of product TE 0.8:1

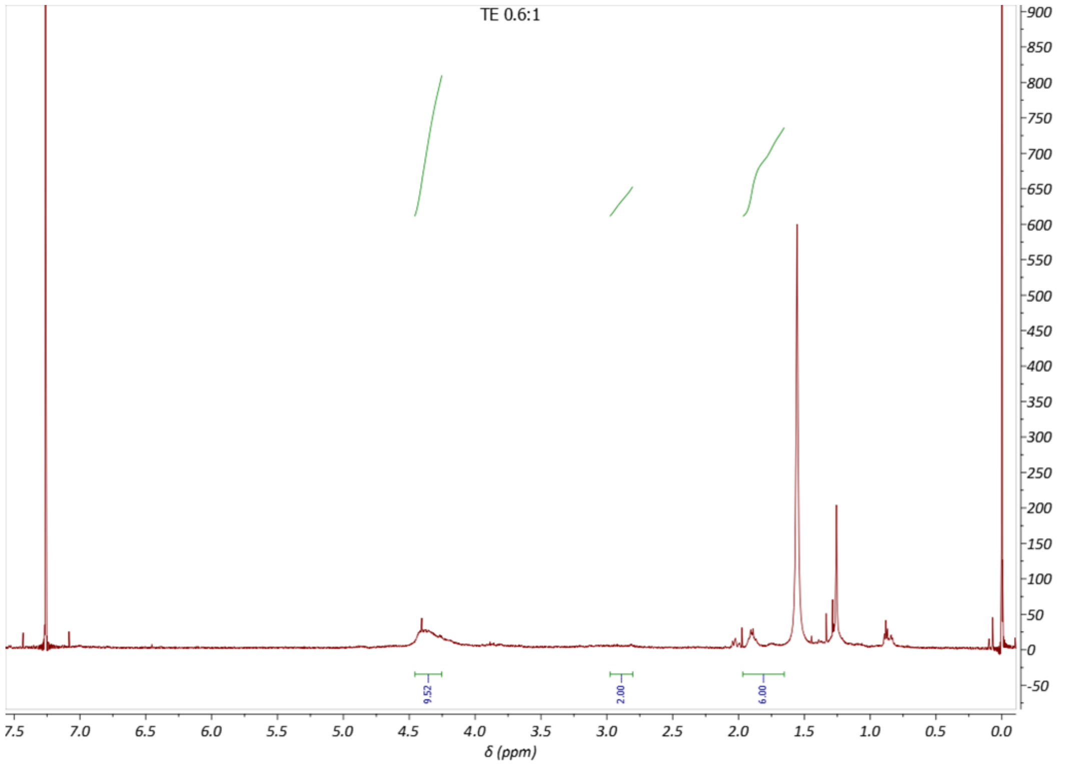

Figure S9. ^{1}H NMR spectrum of product TE 0.6:1

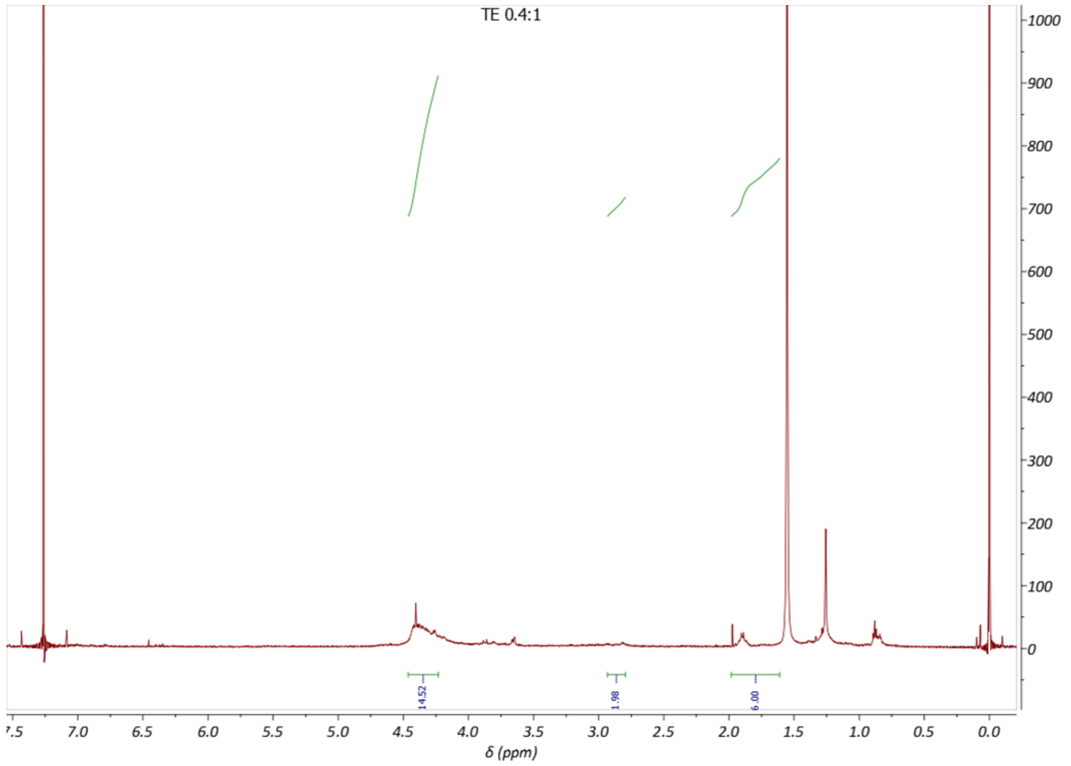

Figure S10. ^{1}H NMR spectrum of product TE 0.4:1

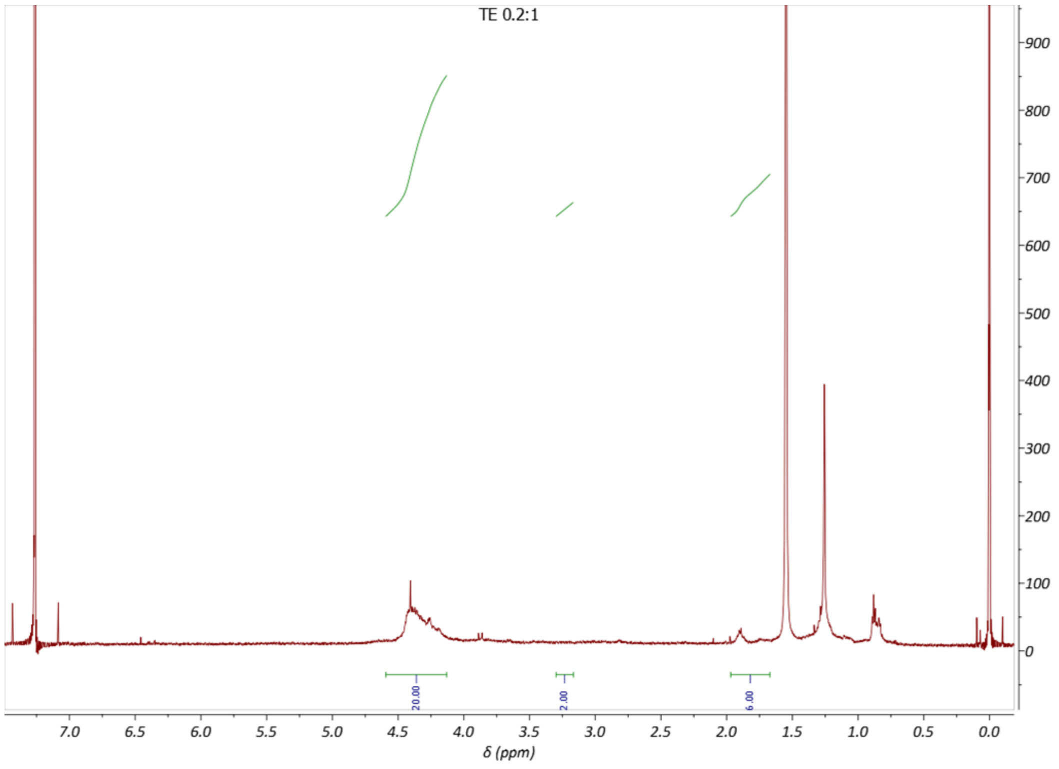

Figure S11. ¹H NMR spectrum of product TE 0.2:1

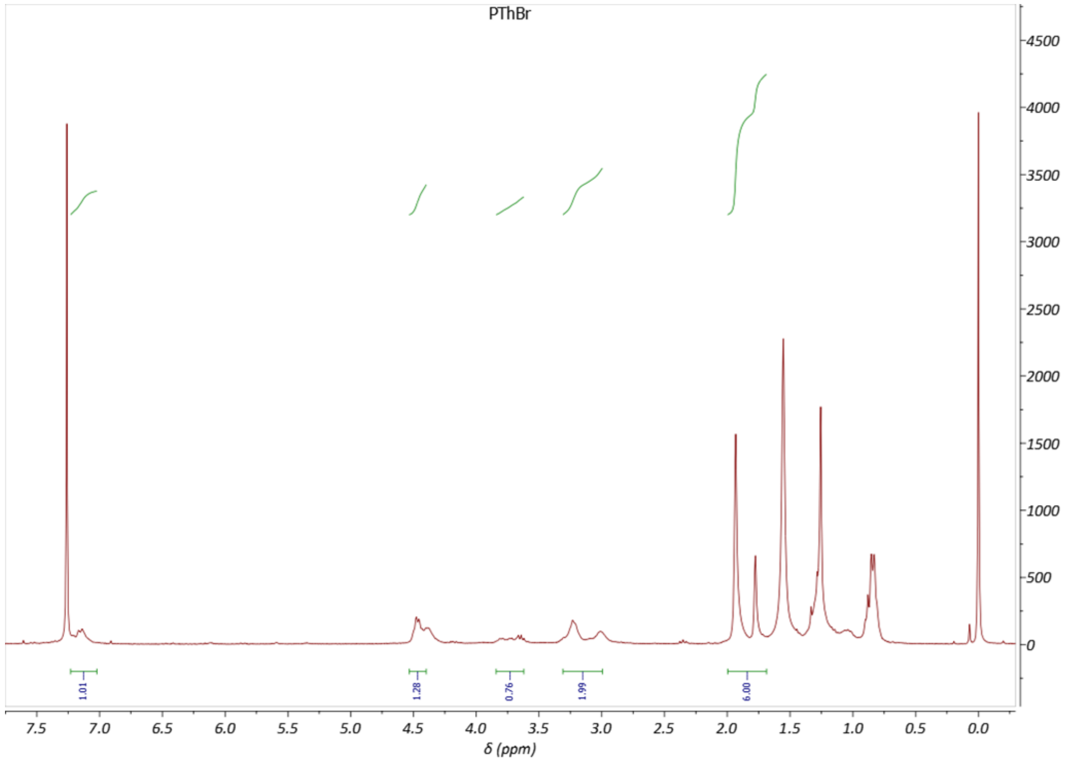

Figure S12. ¹H NMR spectrum of PThBr
